# The Operative management in Bariatric Acute abdomen (OBA) Survey: long-term complications of bariatric surgery and the emergency surgeon’s point of view

**DOI:** 10.1186/s13017-019-0281-y

**Published:** 2020-01-06

**Authors:** Belinda De Simone, Luca Ansaloni, Massimo Sartelli, Yoram Kluger, Fikri M. Abu-Zidan, Walter L. Biffl, Arianna Heyer, Federico Coccolini, Gian Luca Baiocchi, the OBA trial supporters, Fabio Cesare Campanile, Dente Mario, Asnat Raziel, Alberto Zaccaroni, Antonio Tarasconi, Alessandro Dazzi, Konstantinos Lasithiotakisi, Stefano Maccatrozzo, Orestis Ioannidis, Francesco Pata, Maciej Walędziak, Agron Dogjani, Affirul Chairil Ariffin, Vladimir Khokha, Ling Kho, Boris Kessel, Ionut Negoi, Eftychios Lostoridis, Luigi Conti, Luca Ponchietti, Francesco Saverio Papadia, Giorgio Ghilardi, Valentin Calu, Tuna Bilecik, Berhanu N. Alemu, Martin Reichert, Martin Hutan, Charalampos Seretis, Moldovanu Radu, Carlos Augusto Gomes, Faruk Karateke, Vinicius Cordeiro da Fonseca, Arda Isik, Ioannis Nikolopoulos, Anfrii Fomin, Wagih Ghnnam, Ruslan Sydorchuk, Ciro Paolillo, Andrea Sagnotta, Leonardo Solaini, Robert G. Sawyer, David A. Spain, Michael McFarlane, Catherine Arvieux, Andrey Litvin, Bruno M. Pereira, Teresa Giménez Maurel, Andrea Barberis, Mahir Gachabayov, Donal B. O’Connor, Daniel Rios Cruz, Elif Colak, Cristian Mesina, Ramiro Manzano-Nunez, Tadeja Pintar, Renol Koshy, Georgios Triantos, Subash Gautam, Ali Guner, Zaza Demetrashvili, Carlos A. Ordoñez, Dimitrios K. Manatakis, Massimo Chiarugi, Luis Buonomo, Piotr Major, Hecker Andreas, Derek J. Roberts, Jill R. Cherry-Bukowiec, Koray Das, Andrea Costanzi, Harry van Goor, Kenneth Y. Y. Kok, Aleix Martínez-Pérez, Roberta Villa, Luca Fattori, Gabriela Elisa Nita, Dimitrios Damaskos, Kuo-Ching Yuan, Aintzane Lizarazu, Michael Denis Kelly, Isidoro Di Carlo, Marco Ceresoli, Raffaele Galleano, Christos Chatzakis, Miklosh Bala, Frank Piscioneri, Stefano Bonilauri, Helmut Alfredo Segovia Lohse, Dmitry Smirnov, Dennis Kim, Francesca Martina, Giovanni Bruni, Giampiero Campanelli, Marta Cavalli, Victor Kong, Nickos Michalopoulos, Yunfeng Cui, Michele Diana, Fausto Catena

**Affiliations:** 1 Department of General and Emergency Surgery, Azienda Usl Reggio Emilia IRCCS, Reggio Emilia, Italy; 2 Department of Emergency and Trauma Surgery, Bufalini Hospital, Cesena, Italy; 3 Department of General Surgery, Macerata’s Hospital, Macerata, Italy; 4 Department of Emergency and Trauma Surgery, Rambam Health Campus, Haifa, Israel; 5 Department of Surgery, College of Medicine and Health Sciences, UAE University, Al-Ain, United Arab Emirates; 6 Department of Trauma and Acute Care Surgery, Scripps Memorial Hospital, La Jolla, California USA; 7 Sidney Kimmel Medical College, Thomas Jefferson University, Philadelphia, Pennsylvania USA; 8 Department of Surgery, Pisa University Hospital, Pisa, Italy; 9 Department of Surgery, University of Brescia, Brescia, Italy; 10 Department of Emergency and Trauma Surgery, Parma University Hospital, Parma, Italy

**Keywords:** Complication bariatric surgery, Outcome bariatric surgery, Emergency surgery, Acute abdomen, Abdominal pain after bariatric surgery

## Abstract

**Background:**

The number of bariatric procedures is increasing worldwide. No consensus or guidelines about the emergency management of long-term complications following bariatric surgery are currently available. The aim of this study is to investigate by a web survey how an emergency surgeon approaches this unique group of patients in an emergency medical scenario and to report their personal experience.

**Method:**

An international web survey was sent to 197 emergency surgeons with the aim to collect data about emergency surgeons’ experience in the management of patients admitted in the emergency department for acute abdominal pain after bariatric surgery. The survey was conceived as a questionnaire composed by 26 (multiple choice and open) questions and approved by a steering committee.

**Results:**

One hundred seventeen international emergency surgeons decided to join the project and answered to the web survey with a response rate of 59.39%.

**Conclusions:**

The aim of this WSES web survey was to highlight the current management of patients previously submitted to bariatric surgical procedures by ES.

Emergency surgeons must be mindful of postoperative bariatric surgery complications. CT scan with oral intestinal opacification may be useful in making a diagnosis if carefully interpreted by the radiologist and the surgeon.

In case of inconclusive clinical and radiological findings, when symptoms fail to improve, surgical exploration for bariatric patients presenting acute abdominal pain, by laparoscopy if expertise is available, is mandatory in the first 12–24 h, to have good outcomes and decrease morbidity rate.

## Background

The World Health Organization (WHO) reported that the worldwide prevalence of obesity nearly tripled between 1975 and 2016. There are 340 million children and adolescents (age 5–19) who are overweight or obese. In 2016, more than 1.9 billion adults aged 18 years and older were overweight. Of these, over 650 million adults were obese. Overall, about 13% of the world’s adult population (11% of men and 15% of women) were obese in 2016 [1].

Morbid obesity occurs in 2–5% of the Western population and is associated with a high incidence of multiple preventable co-morbidities such as diabetes, cancer, and cardiovascular disease. Morbid obesity increases the risk of mortality [1].

Bariatric surgery is the only method that has been shown to achieve long term weight loss and treat co-morbidities [2].

The number of bariatric procedures performed by bariatric surgeons is increasing in specialist centers and abroad due to the phenomenon of health tourism [2–3]. The most recent International Federation for the Surgery of Obesity and metabolic Disorders (IFSO) Worldwide Survey [3] reported that 634,897 bariatric operations were performed worldwide in 2016.

The IFSO worldwide survey 2014 reported that the current most commonly performed bariatric procedures are the sleeve gastrectomy (SG), Roux en-Y gastric bypass (RYGB) and laparoscopic adjustable gastric band (LAGB). These procedures represent respectively 45.9, 39.6, and 7.4% of all bariatric procedures performed worldwide. RYGB is the most common bariatric surgery in the UK followed by SG, although the latter has been gaining in popularity and is now the most common bariatric surgery in countries where most bariatric surgeries are performed such as other European and North American countries. A total of 6391 bariatric surgical procedures were performed in the UK in 2014 compared with 191,920 in the USA and 46,960 in France [3–4].

The number of bariatric procedures performed is increasing, leading to more post-operative bariatric patients that will present with an acute abdomen in the emergency department.

Patients with early postoperative complications may be managed in specialist centers by the bariatric surgeon during the hospital stay but patients with acute abdominal pain that occurs after months or years post-operatively may present for assessment and management in the local emergency units.

Complications following surgical treatment of severe obesity vary based upon the procedure performed and can be as high as 40% [4]. Due to the wide variety of surgical bariatric techniques, the functional outcomes and late or long-term complications (those that occur after 1 month after surgery) from bariatric surgery remain not completely known or well understood.

No consensus or guidelines about the emergency management of long-term complications following bariatric surgery are currently available.

The aim of this study is to investigate by a web survey how an emergency surgeon approaches this unique group of patients in an emergency medical scenario and to report their experience.

## Method

This study reports data collected by an international web survey carried out with the aim to collect data about emergency surgeons’ experience in the management of patients admitted in the emergency department for acute abdominal pain (AA) after bariatric surgery.

The survey was conceived as a questionnaire composed by 26 (multiple choice and open) questions and was sent on January 28, 2018, via Google Forms, after the approval of the World Society of Emergency Surgery (WSES) project steering committee represented by Fausto Catena (Parma Trauma Center, Italy), Luca Ansaloni (Cesena Trauma Center, Italy), Yoram Kluger (Rambam Health Care Center, Israel), and Walter L. Biffl (Scripps Clinic, San Diego, USA) to the mailing list of the WSES members.

The deadline to participate was March 28, 2018.

The project’s main objectives were the following:

1) To extrapolate epidemiological characteristics and clinical-pathological features about this population of patients admitted to the emergency department for acute abdominal pain;

2) To highlight life-threatening complications and outcomes of bariatric surgery;

3) To analyze the decision-making algorithms of the emergency surgeons in the management of AA in patients previously treated with bariatric surgical procedures to determine best practices for early diagnosis, and best operative and non-operative treatments to decrease morbidity and in-hospital mortality rates.

## Results

The invitation to participate in the web survey was sent to 197 surgeons.

One hundred seventeen international emergency surgeons (ES) decided to join the project and answered to the web survey with a response rate of 59.39%.

**Fig. 1:**
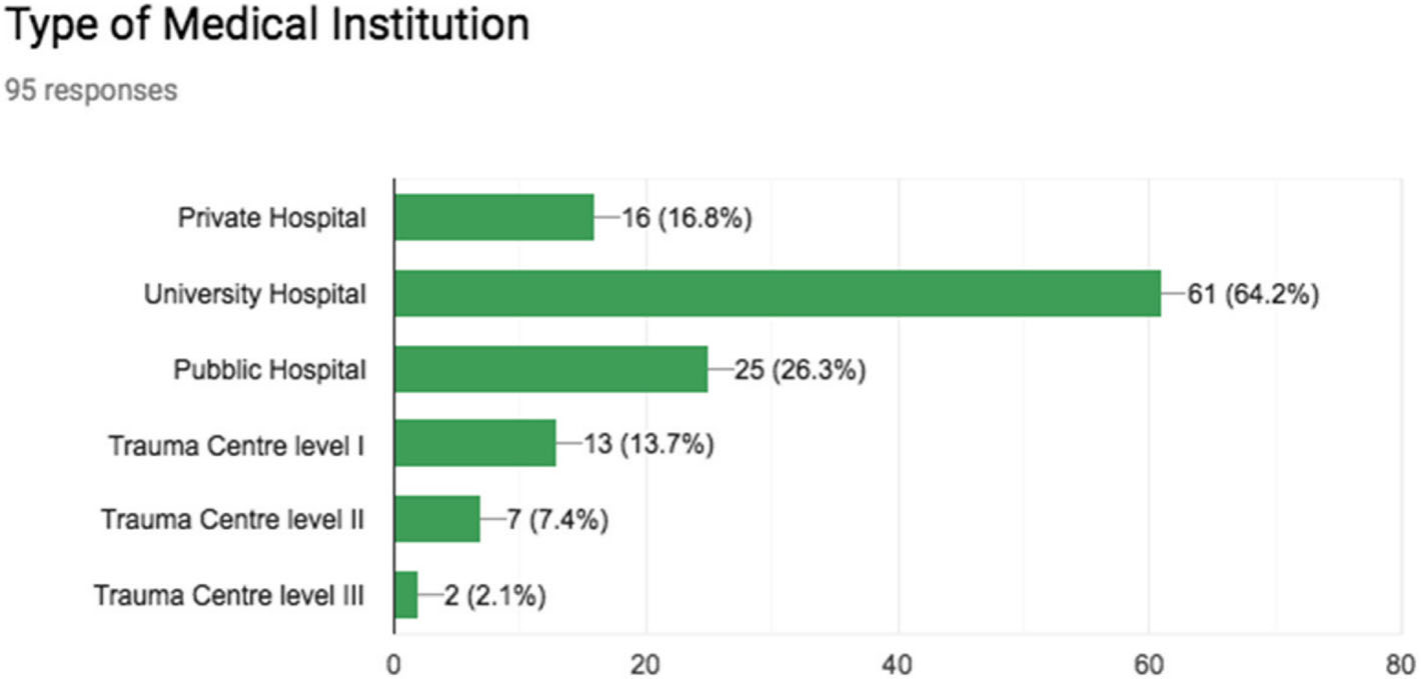
Participants’ affiliations

Sixty-four percent (61/95) of ES worked in a university hospital, 26.31% (25/95) in a public hospital, 16.8% (16/95) in a private hospital and 13.6% (13/95) in a trauma center level I, 7.4% (7/95) in a trauma center level II, 2.1% (2/95) in a trauma center level III as summarized in Fig. 1

Most participants (51.8.%; 68/117 ES) declared to have over 10 years of surgical experience and 25.6% (30/117) have surgical experience of 5 to 10 years.

The majority of ES (55.6%; 65/117) work in a hospital with a bariatric unit and almost all (97.4%; 114/117 ES) in a hospital with an intensive care unit (ICU). 59.5% of responders (69/117) declared to have no experience in bariatric procedures, but almost all surgeons (98.3%; 115/117) have been called to evaluate an AA after bariatric surgery in an emergency department (ED). The majority of ES reported to have managed less than 10 bariatric patients in their experience (52%; 61/117), 24% (29/117) between 10 and 20 bariatric patients, and 23% (27/117) more than 20 patients.

According to the answers, 36.8% (43/117) of bariatric patients examined presented with AA after less than 4 weeks from the bariatric surgical procedure, 22.2% (26/117) between 4 weeks and 6 months, 16.2% (19/117) between 6 months and 1 year, and 25% (29/117) after over 1 year following bariatric surgery. The majority of patients were female (76.7%; 91/117) over 40 years old (59.8%; 70/117), and capable of reporting their surgical history and specific type of bariatric surgical procedure previously performed (77%; 91/117).

Most of the examined patients (44/117; 37.6%) had been operated on in the same hospital as that of the ES on call, while 32.5% (38/117) were operated in a private hospital, 28% (33/117) in another public hospital and 1.7% (2/117) were operated on in a different country.

The majority of patients had received a sleeve gastrectomy (38.5%; 45/117), and 31.6% (37/117) a laparoscopic Roux-en-Y gastric bypass as summarized in Table 1.

**Table 1:** Type of bariatric surgery previously undergone by patient presenting with acute abdominal pain

Type of bariatric surgery	Number of answers	%
Sleeve gastrectomy	45/117	38.5
Laparoscopic Roux en Y gastric bypass	37/117	31.6
Open Roux en Y gastric bypass	4/117	3.4
Unknown	9/117	7.7
Laparoscopic adjustable gastric binding	22/117	18.8

**Fig. 2:**
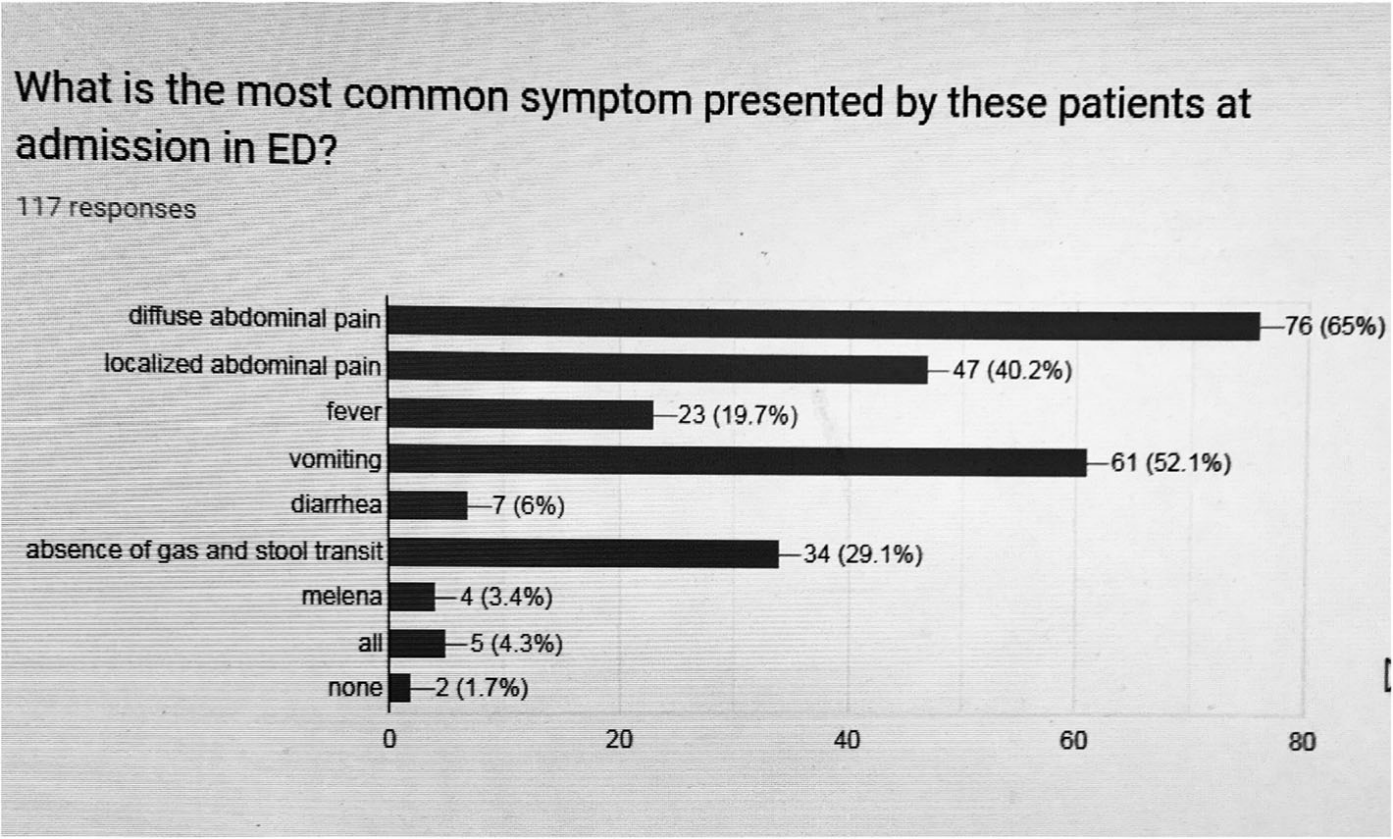
Most common symptoms presented by bariatric patients admitted in emergency department

The most common complaint was generalized abdominal pain (65%; 76/117), followed by vomiting (52%; 61/117) and localized abdominal pain (40.2%; 47/117) as summarized in Fig. 2.

In evaluating the patients, 37.6% (44/117) of the ES asked for the following diagnostic laboratory exams, as summarized in Table 2: complete blood count (CBC), dosage of electrolytes, protein C-reactive (PCR), and/or procalcitonin (PCT).

**Table 2:** Common laboratory tests requested at admission of bariatric patients

Laboratory tests requested	Number of answers	%
CBC, dosage electrolytes, dosage CPR, and/or PCT	45/117	38.4
CBC, blood gas analysis, lactates, CPR, and/or PCT	39/117	33.33
CBC, liver function tests, dosage lipase, dosage troponin, dosage CPR, and/or PCT	33/117	28.2

Eighty-seven/117 (74.4%) of ES reported that laboratory exams were a useful diagnostic tool, and 30/117 (25.6%) of ES reported that they were not.

Radiological exams performed to aid in diagnosis included plain abdominal radiography and enhanced computed tomography (CT) in 41.9% of responses (49/117), abdominal CT with intestinal opacification in 41.9% of responses (49/117), and plain abdominal radiography in standing position and abdominal US, in 13.7% of responses (16/117), as summarized in Table 3.

**Table 3:** Common radiological exams requested to evaluate acute abdomen in bariatric patients

Radiological exams requested	Number of answers	%
Abdominal CT scan with oral intestinal opacification	49/117	41.9
Plain XR, enhanced CT scan	49/117	41.9
Plain XR, US	16/117	13.6
Plain XR	1/117	0.85
UGI, CT	1/117	0.85

Radiological exam results were useful in the decision-making of 109/117 ES (93.2%).

**Fig. 3:**
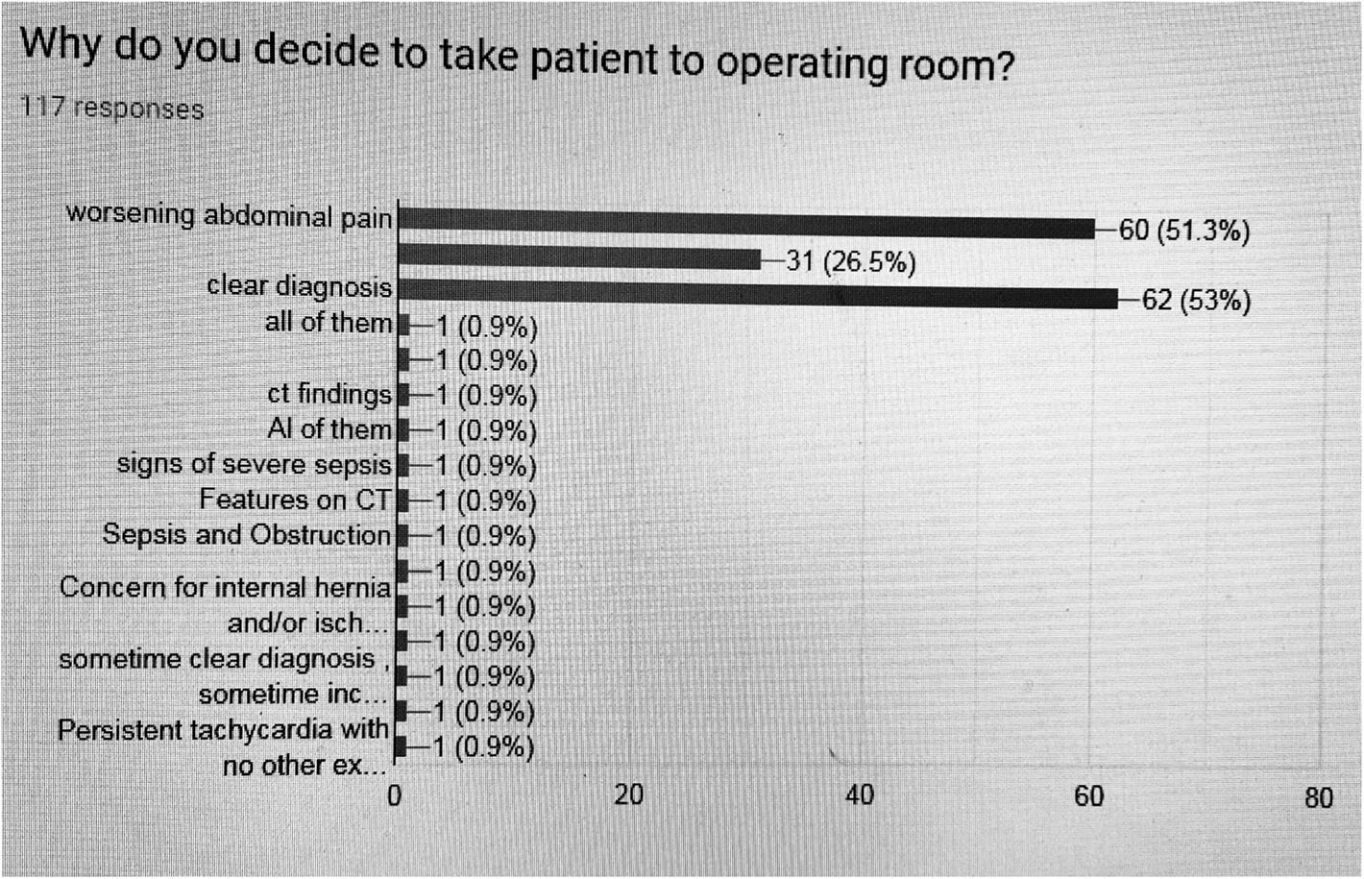
Why emergency surgeons decide to take the bariatric patient into the operating room

62/117 (53%) of ES took patients to the operating room because of a clear diagnosis, 60/117 (51.3%) of ES because of worsening abdominal pain, and 31/117 (26.5%) of ES for inconclusive findings as summarized in Fig. 3.

Timing for surgery was between 12 and 24 h in 51/117 responses (43.5%), < 12 h for 41.9% (49/117) of responses, > 24 h for 12.8% (15/117) of responses, variable according to diagnosis for 2/117 (1.7%) of responses as summarized in Table 4.

**Table 4:** Delay from admission to operating room

Delay	Number of answers	%
< 12 h	50/117	42.73
12–24 h	49/117	41.9
> 24 h	15/117	12.8
< 24 h	1/117	0.85
Variable according to diagnosis	2/117	1.7

*UGI* upper gastrointestinal series *CT* abdominal computed tomography, *US* ultrasonography, *XR* X-ray

Surgical exploration was performed by laparoscopy in more than 50% of bariatric patients for 57/117 of ES (48.7%), by laparoscopy in less than 50% of cases for 24/117 (20.5%) of ES, by laparotomy in more than 50% of cases for 19/117 (16.2%) of ES, by laparotomy in all cases for 16/117 surgeons (13.7%), by laparotomy in less than 50% of cases for 1/117 ES (0.9%) as summarized in Table 5.

**Table 5:** Technique for surgical emergency exploration in patients presenting acute abdomen previously submitted to bariatric surgery

Technique for surgical emergency exploration	Number of answers	%
Laparoscopy in < 50% of cases	24/117	20.5
Laparoscopy in > 50% of cases	57/117	48.7
Laparotomy in all cases	16/117	13.7
Laparotomy in < 50% of cases	1/117	0.85
Laparotomy in > 50% of cases	19/117	16.2

Intra-operative findings reported were summarized in Table 6.

**Table 6:** Common intra-operative findings in bariatric patients

Intraoperative findings	Number of answers	%
Internal hernia	58/117	49.5
Adhesions	49/117	41.8
Anastomotic stenosis	15/117	12.8
Intussusception	9/117	7.6
Volvulus	9/117	7.6
Leak	5/117	4.2
Complications of gastric band	4/117	3.4
Gastric perforation	1/117	0.85
Hemorrhagic ulcer in esclude stomach	1/117	0.85
Peritonitis	3/117	2.5
Leaking stapler line	1/117	0.85
Cholecystitis	1/117	0.85
Bleeding, abscesses	1/117	0.85
Perforation	1/117	0.85
Bleeding, mesenteric thrombosis	1/117	0.85

In-hospital mortality rate reported was < 10% in 69.2% (81/117) of answers, between 10 and 50% for 19/117 (16.2%) of ES, “low” for 1 surgeon (0.9%), and unknown for 16/117 (13.7%) of ES.

Fifty-six/117 (47.9%) of ES reported that their patients required admission to the ICU after surgery; 15/117 (12.8%) of ES reported that theirs did not; and 46/117 (39.3%) of ES answered “maybe.”

Most ES, 112/117 (95.7%), reported that their patients were discharged alive.

72.6% (85/117) of ES declared to be worried about bariatric patients presenting with AA.

## Discussion

The present international survey was conceived to assess knowledge and clinical practice about the management of AA in patients previously submitted to bariatric surgery, in an emergency setting. 59.3% of invited ES decided to join the project, confirming the increasing interest to explore this topic, especially in light of the current lack of consensus and guidelines.

The quality of data collected by this questionnaire derives from the seniority (51.8% of respondents declared to have a surgical practice of more than 10 years) and the internationality of the respondents.

The survey reported that not all ES have experience in bariatric surgery and not all hospitals have a bariatric surgery unit, consequently bariatric patients needing re-intervention for acute abdomen were managed by the ES on call.

Late complications following bariatric surgery have been poorly analyzed and their management is not clearly assessed in the emergency setting.

Collected data showed that most of the bariatric patients (BP) admitted in the ED were female, mean age of greater than 40 years and presenting with acute generalized abdominal pain (65% of answers) and vomiting (52.1% of answers) within 4 weeks after the surgical intervention.

The survey showed that SG was the most commonly reported surgical procedure (38.5%), followed by LRYGB (31.6%).

Clinical signs and physical examination of BP presenting with AA can be atypical, insidious, often resulting in delayed management due to inconclusive clinical and radiological findings, with poor outcomes and high morbidity rate. Tachycardia is considered the alarm sign for all bariatric surgeons in the early postoperative time. Late complications can be revealed by hemodynamic instability, respiratory failure or renal dysfunction. However, these are not always present.

Several studies confirmed that abdominal pain is one of the most common and sometimes frustrating problems after bariatric surgery and some authors affirmed that anywhere from 15 to 30% of patients will visit the emergency room or require admission within 3 years of gastric bypass [5–8].

In particular, Saunders et al. **[**7**]** reported that the overall 1-year readmission rate for abdominal pain in a high volume bariatric center was 18.8% and that most of the patients were re-admitted after LRYGB (24.2%), whereas LAGB showed the lowest readmission rate of 12.69%.

Another study confirmed this data showing that the ≤ 90-day all-cause postoperative ED visit rate was 18% (65/361 BP) in a bariatric center [6].

The most common postoperative complications of bariatric procedures described in literature are summarized in Table 7 [9–16].

**Table 7:** Common complications following bariatric surgery

Bariatric surgical procedures	Early complications	Late complications
Sleeve gastrectomy	Leak/fistula	Gastroesophageal reflux
	stricture	
	hemorrhage	
Gastric bypass	Leak/fistula	Anastomotic ulcer (bleeding, perforation)
	obstruction/anastomotic	
	stricture	bowel obstruction (internal hernia)
	hemorrhage	
Adjustable gastric binding	Esophageal and/or gastric perforation	Infection
		connector tubing rupture
	acute dilatation of the gastric pouch	gastric pouch dilatation and slippage of the AGB
		erosion and intragastric migration
		esophageal dilatation

Complication rates are reported higher after LRYGB but we cannot confirm that: most surgeons reported evaluating patients presenting with abdominal pain after SG [11–14].

In agreement with available studies [17–23], the WSES survey reported that ES used enhanced abdominal computed tomography (CT) with oral intestinal opacification to make a diagnosis in BP, even if only 53% of ES declared that diagnosis after radiological exams was clear.

Diagnostic value of imaging in BP depends on the careful interpretation of the new anatomical landmarks and on the knowledge of the potential complications following bariatric surgery.

Several studies described the new radiological anatomy after bariatric surgery at CT scan. The administration of oral and intravenous contrast is fundamental to find landmarks for the interpretation of images [19–23]. For example, after gastric bypass, the identification of the gastric pouch, gastrojejunal anastomosis, jejunal Roux limb, jejunojejunal anastomosis, and biliopancreatic limb on CT is essential for detecting potential complications such as internal hernias and small bowel obstruction (SBO). Positive oral contrast material administered just prior to image acquisition helps differentiate the gastric pouch and Roux limb from the excluded stomach and biliopancreatic limb, which are not opacified. The Roux limb should be followed along its antecolic or retrocolic course to the jejunojejunal anastomosis, typically in the left mid-abdomen. The excluded stomach should be visualized on CT images and is normally collapsed [19, 20].

According to CT scan findings, SBO following RYGB is classified on the features of the Roux-alimentary limb, bilio-pancreatic limb and distal common channel involvement [18].

After SG, CT scan is the right radiological exam to assess for abscesses, perforation, staple line dehiscence, and other complications such as splenic injury or infarction [19, 20].

Our survey reported that internal hernia (49.6% of answers) and adhesions (41.9% of answers) were common intraoperative findings at surgical exploration (Table 6), suggesting that SBO is the leading cause of abdominal pain after bariatric surgery.

SBO occurs in approximately 5% of cases after gastric bypass and is due frequently to adhesions or to internal herniation. Other causes of SBO are incisional hernia through a trocar opening or intussusception of the small bowel [21].

Internal herniation occurs in approximately 6% of patients after gastric bypass or biliary pancreatic shunting and it can be a potentially fatal complication [22, 23].

It is promoted by massive weight loss and by characteristic mesenteric defects that develop after LRYGB that are in the transverse mesocolon for a retrocolic Roux limb, a mesenteric defect near the jejunojejunal anastomosis and a defect posterior to the Roux limb (i.e., Petersen’s defect).

Internal hernias are very difficult to reveal clinical inquiry and from radiological investigations and requires a high index of suspicion. The sensitivity of CT scan in identifying the “mesenteric swirl sign,” the most sensitive CT scan sign suggestive of internal hernia, has been reported to be between 68 and 89% [17].

Anastomotic stenosis can cause an obstruction and it usually involves the gastrojejunal anastomosis. It occurs in approximately 12% of patients after bypass and typically develops a month or more after surgery with a peak occurring 50 days after gastric bypass [16, 17].

Patients presenting with bariatric surgery complications in an emergency setting have a poor outcome, largely related to delayed diagnosis and re-operation, but no data are available.

Our survey showed that in-hospital mortality related to re-operated BP is < 10% for 69.2% of ES and that the majority of patients were discharged alive (95.7% of answers).

Diminishing the delay in surgery is crucial to avoid catastrophic scenarios such as generalized peritonitis due to intestinal perforation or massive bowel ischemia.

Our survey reported that the majority of ES do not wait for more than 24 h to decide in favor of surgical exploration if the patient presents with worsening abdominal pain and inconclusive radiological findings (Fig. 3).

Our data showed that surgical exploration was made by laparoscopy for the majority of ES, in more than 50% of BP.

This is in accordance with several available studies that investigated the role of explorative laparoscopy to assess chronic abdominal pain after bariatric surgery. These studies demonstrated that the laparoscopic approach is safe and feasible in BP presenting with abdominal pain of unknown etiology [24, 25].

All of these studies express concerns regarding chronic pain in BP and the diagnostic value of explorative laparoscopy. Other (case reports and retrospective studies) authors reported data about the management of AA after bariatric surgery by laparoscopy confirming that laparoscopy is feasible and safe even in the emergency setting, if expertise is available and the patient is hemodynamically stable [26–28].

Despite a good correspondence between the data resulting from our survey and the current data available in literature about the management of acute abdomen in bariatric patients, 85/117 (72.6%) of ES declared to be worried when asked to manage acute abdomen in patients with a previous history of bariatric surgery. This indicates the ES’ desire to be familiar with the various types of bariatric surgery, to understand the new anatomy, radiological findings, and long-term bariatric complications, to be able to appropriately manage them in the emergency setting.

We acknowledge the limitations of the present study, some due to the intrinsic nature of surveys (answers may not be honest and accurate, responders represent an intrinsic selection bias because non-responders may answer differently, answer options may be interpreted differently by different responders), and some related to the nature of our data, not linked to a population of patients but to the personal experience and opinion of 117 international ES.

## Conclusions

Bariatric procedures are increasing and this results in an increased number of bariatric patients admitted in the ED for AA. ES have a crucial role in the management of this group of patients and no consensus or guidelines are available.

The aim of this WSES web survey was to highlight the current management of bariatric patients in the ED by ES.

Emergency surgeons must be mindful of postoperative bariatric surgery complications. CT scan with oral intestinal opacification may be useful in making a diagnosis if carefully interpreted by the radiologist and the surgeon.

In the case of inconclusive clinical and radiological findings, when symptoms fail to improve, early surgical exploration, by laparoscopy if expertise is available, is mandatory in the first 12–24 h, to have good outcomes and decrease morbidity rate.

## Data Availability

Not applicable

## Abbreviations

AA: Acute abdomen
BP: Bariatric patient(s)
CT: Abdominal computed tomography
ED: Emergency department
ES: Emergency surgeon(s)
LAGB: Laparoscopic adjustable gastric binding
RYGB: Roux en Y gastric bypass
SBO: Small bowel obstruction
SG: Sleeve gastrectomy
