# Correction to: The Operative management in Bariatric Acute abdomen (OBA) Survey: long-term complications of bariatric surgery and the emergency surgeon’s point of view

**DOI:** 10.1186/s13017-020-0292-8

**Published:** 2020-02-04

**Authors:** Belinda De Simone, Luca Ansaloni, Massimo Sartelli, Yoram Kluger, Fikri M. Abu-Zidan, Walter L. Biffl, Arianna Heyer, Federico Coccolini, Gian Luca Baiocchi, the OBA trial supporters, F. C. Campanile, D. Mario, A. Raziel, A. Zaccaroni, A. Tarasconi, A. Dazzi, K. Lasithiotakisi, S. Maccatrozzo, O. Ioannidis, F. Pata, M. Walędziak, A. Dogjani, A. C. Ariffin, V. Khokha, L. Kho, B. Kessel, I. Negoi, E. Lostoridis, L. Conti, L. Ponchietti, F. S. Papadia, G. Ghilardi, V. Calu, T. Bilecik, B. N. Alemu, M. Reichert, M. Hutan, C. Seretis, M. Radu, C. A. Gomes, F. Karateke, V. C. da Fonseca, A. Isik, I. Nikolopoulos, A. Fomin, W. Ghnnam, R. Sydorchuk, C. Paolillo, A. Sagnotta, L. Solaini, R. G. Sawyer, D. A. Spain, M. McFarlane, C. Arvieux, A. Litvin, B. M. Pereira, T. G. Maurel, A. Barberis, M. Gachabayov, D. B. O’Connor, D. R. Cruz, E. Colak, C. Mesina, R. Manzano-Nunez, T. Pintar, R. Koshy, G. Triantos, S. Gautam, A. Guner, Z. Demetrashvili, C. A. Ordoñez, D. K. Manatakis, M. Chiarugi, L. Buonomo, P. Major, H. Andreas, D. J. Roberts, J. R. Cherry-Bukowiec, K. Das, A. Costanzi, H. van Goor, K. Y. Y. Kok, A. Martínez-Pérez, R. Villa, L. Fattori, G. E. Nita, D. Damaskos, K. C. Yuan, A. Lizarazu, M. D. Kelly, I. D. Carlo, M. Ceresoli, R. Galleano, C. Chatzakis, M. Bala, F. Piscioneri, S. Bonilauri, H. A. S. Lohse, D. Smirnov, D. Kim, F. Martina, G. Bruni, G. Campanelli, M. Cavalli, V. Kong, N. Michalopoulos, Y. Cui, M. Diana, Fausto Catena

**Affiliations:** 1 Department of General and Emergency Surgery, Azienda Usl Reggio Emilia IRCCS, Reggio Emilia, Italy; 2 Department of Emergency and Trauma Surgery, Bufalini Hospital, Cesena, Italy; 3 Department of General Surgery, Macerata’s Hospital, Macerata, Italy; 4 Department of Emergency and Trauma Surgery, Rambam Health Campus, Haifa, Israel; 5 Department of Surgery, College of Medicine and Health Sciences, UAE University, Al-Ain, United Arab Emirates; 6 Department of Trauma and Acute Care Surgery, Scripps Memorial Hospital, La Jolla, California USA; 7 Sidney Kimmel Medical College, Thomas Jefferson University, Philadelphia, Pennsylvania USA; 8 Department of Surgery, Pisa University Hospital, Pisa, Italy; 9 Department of Surgery, University of Brescia, Brescia, Italy; 10 Department of Emergency and Trauma Surgery, Parma University Hospital, Parma, Italy

**Correction to: World J Emerg Surg (2020) 15:2**

**https://doi.org/10.1186/s13017-019-0281-y**

The original article [1] contained an error in authorship whereby author, Fausto Catena was mistakenly listed as part of the institutional authorship of the OBA trial supporters instead of in the correct position of final author.

As such, the original article has since been corrected to reflect the correct authorship.

Furthermore, this error was mistakenly introduced by the production team handling this article and, as such was not the fault of the authors.

## References

[1] De Simone B, Ansaloni L, Sartelli M, Kluger Y, Abu-Zidan FM, Biffl WL (2020). The Operative management in Bariatric Acute abdomen (OBA) Survey: long-term complications of bariatric surgery and the emergency surgeon’s point of view. World J Emerg Surg.

